# Social Networks Reshape the Impact of Elder Abuse on Social Well-being: Examining Urban-Rural Disparities in China

**DOI:** 10.1093/geroni/igaf122.3143

**Published:** 2025-12-31

**Authors:** Xin Sun, Zi Yan

**Affiliations:** Fudan University, Shanghai, Shanghai, China (People’s Republic); Waseda University, Tokyo, Tokyo, Japan

## Abstract

The contextual theory of elder abuse emphasizes that elder abuse arises from the interaction of individual, relational, community, and societal contexts. This study tests this theory using nationally longitudinal data from 7,125 older adults (aged ≥60 years) in the 2018 and 2020 China Longitudinal Ageing Social Survey. We regressed changes in social networks, and social well-being in 2020 on elder abuse experience in 2018 and found that the victims have better social well-being two years later than those who did not report abuse. A novel association was identified between elder abuse, reduced contact, and emotional networks, and improved social well-being among rural victims. Conversely, among urban victims, elder abuse was associated with an expanded instrumental network and improved social well-being. Our findings point to the transforming impact of social networks on elder abuse victims’ social well-being. These findings underscore the importance of considering urban-rural disparities in designing culturally adaptive interventions and support systems that leverage social networks to buffer the adverse effects of elder abuse.

